# Peripheral blood cellular dynamics of rheumatoid arthritis treatment informs about efficacy of response to disease modifying drugs

**DOI:** 10.1038/s41598-023-36999-0

**Published:** 2023-06-21

**Authors:** Åsa K. Hedman, Eitan Winter, Niyaz Yoosuf, Yair Benita, Louise Berg, Boel Brynedal, Lasse Folkersen, Lars Klareskog, Mateusz Maciejewski, Alexandra Sirota-Madi, Yael Spector, Daniel Ziemek, Leonid Padyukov, Shai S. Shen-Orr, Scott A. Jelinsky

**Affiliations:** 1 Division of Rheumatology, Department of Medicine Solna, Karolinska Institutet and Karolinska University Hospital, Stockholm, Sweden; 2 Center for Molecular Medicine, Karolinska Institutet, Stockholm, Sweden; 3 Institute of Environmental Medicine, Karolinska Institute, Stockholm, Sweden; 4 Department of Inflammation and Immunology, Pfizer, 1 Portland Street, Cambridge, MA 02139 USA; 5 Department of Inflammation and Immunology, Pfizer, Berlin, Germany; 6 CytoReason, Tel-Aviv, Israel; 7 Technion-Israel Institute of Technology, Haifa, Israel

**Keywords:** Computational biology and bioinformatics, Immunology

## Abstract

Rheumatoid arthritis (RA) is an autoimmune disease characterized by systemic inflammation and is mediated by multiple immune cell types. In this work, we aimed to determine the relevance of changes in cell proportions in peripheral blood mononuclear cells (PBMCs) during the development of disease and following treatment. Samples from healthy blood donors, newly diagnosed RA patients, and established RA patients that had an inadequate response to MTX and were about to start tumor necrosis factor inhibitors (TNFi) treatment were collected before and after 3 months of treatment. We used in parallel a computational deconvolution approach based on RNA expression and flow cytometry to determine the relative cell-type frequencies. Cell-type frequencies from deconvolution of gene expression indicate that monocytes (both classical and non-classical) and CD4^+^ cells (T_h_1 and T_h_2) were increased in RA patients compared to controls, while NK cells and B cells (naïve and mature) were significantly decreased in RA patients. Treatment with MTX caused a decrease in B cells (memory and plasma cell), and a decrease in CD4 T_h_ cells (T_h_1 and T_h_17), while treatment with TNFi resulted in a significant increase in the population of B cells. Characterization of the RNA expression patterns found that most of the differentially expressed genes in RA subjects after treatment can be explained by changes in cell frequencies (98% and 74% respectively for MTX and TNFi).

## Introduction

Rheumatoid arthritis (RA) is an autoimmune disease characterized by chronic inflammation in joints and synovial hyperplasia leading to bone and cartilage destruction. RA can result in progressive disability including arthralgia, swelling, redness, and eventually limiting the range of motion. RA is an autoimmune and inflammatory disease and is strongly associated with the alteration of several immune cell populations^1^ which is directly or indirectly involved in inflammation, generation of pain and joint tissue destruction. Current therapies are aimed at alleviating pain and preventing joint damage^2^. The treatment of RA has evolved significantly over the years, with the advent of various classes of disease-modifying antirheumatic drugs (DMARDs). Conventional synthetic DMARDs (csDMARDs) such as methotrexate (MTX), hydroxychloroquine (HCQ), and sulfasalazine (SSZ) have been the cornerstone of RA management for decades and are currently the first-line therapy for treatment of RA^3^. These drugs act by suppressing the immune system and reducing inflammation, thereby preventing joint damage and improving quality of life for patients. However, not all patients respond to csDMARDs, and some may experience adverse effects. Targeted synthetic DMARDs (tsDMARDs) such as tocilizumab and tofacitinib, have specific molecular targets including IL-6^4^ and JAK kinases^5^ and biologic DMARDs (bDMARDs) such as tumor necrosis factor inhibitors (TNFi)^6^ have emerged as alternative options for patients who are unresponsive to csDMARDs or cannot tolerate their adverse effects.

MTX is the most commonly used initial treatment for RA, targeting folate-dependent enzymes involved in de novo pyrimidine and purine synthesis. However, the anti-inflammatory effects of MTX are not fully understood. It is known that MTX directly and indirectly regulates the function of many diverse cell types. MTX increases regulatory T-cells, decreases levels of IL-6, reduces TNF produced by T cells^7^, inhibits proliferation cytokine production by monocytes/macrophages^8,9^, and is involved in modulation of function of T cells, B cells, neutrophils, monocytes, and fibroblast-like synoviocytes (FLSs)^10^. Methotrexate inhibits several key enzymes in the folate pathway. This inhibition can lead to a decrease in intracellular levels of folates, which are essential for the de novo synthesis of purines and pyrimidines, and consequently a decrease in nucleotide synthesis. This decrease in nucleotide synthesis can lead to an accumulation of adenosine in the extracellular space, which has been shown to activate adenylate cyclase and increase cAMP levels. Increased cAMP levels can, in turn, have anti-inflammatory effects by inhibiting the production of pro-inflammatory cytokines and promoting the production of anti-inflammatory cytokines including TNF, IFN-γ, and IL-1β^11^. Given its effects on multiple cell types, MTX is considered to be a general immune modulator^12^. Depending on the patients’ condition and response profile to MTX, more targeted therapies are being used to treat RA including TNFi^13^. TNF is a major pro-inflammatory cytokine synthesized mostly by macrophages and T cells. TNF receptors are expressed on nearly every cell type allowing TNF to control many different immune cell types^14^.

Significant effort has been undertaken to understand the molecular mechanisms of disease progression and response to current therapies. mRNA expression analysis by RNAseq and microarray analysis have identified regulated genes and pathways associated with RA^15–21^. In addition, genome-wide association studies (GWAS) approaches (e.g.^22,23^) have reported several associated genes that play key roles in RA pathology.

Gene expression profiling is an informative method used to investigate disease biology. However, changes in cellular composition, a major contributor to gene expression changes, is not systematically studied in transcriptomics studies. To build an accurate molecular profile of a phenotype, cell composition must be accounted for in order to understand the true gene expression regulatory changes. The complexity of the detection of different cell types in tissue samples, including peripheral blood, could be addressed either by direct measure or by imputation from gene expression or DNA methylation data. Direct measure of blood cells by flow cytometry and by cell counting after antibody staining or by cell morphology are common approaches that are usually limited by the number of specific sub-populations detected in flow cytometry experiments. Additionally, flow cytometry-based methods require certain logistical considerations to collect a sample of fresh blood for flow cytometry and simultaneously, a sample for transcriptomics.

Several computational methods to deconvolve cell composition based on gene expression have been developed^24–30^. The development of single-cell RNA-seq (scRNA-seq) technologies has enabled cell type-specific transcriptome profiling. However, scRNA-seq is still unreliable for cell composition measurement since single-cell preparation protocols have different efficiencies for different cell types. For instance, neutrophils, which are the most abundant immune cells in the blood, are poorly represented in scRNAseq analyses. In addition, most of the historical data available relied on bulk gene expression measurements.

To investigate the contribution of immune cells in response to treatment in RA, we employed a novel analytical strategy to profile RA patients compared to healthy controls, as well as pre- to post-treatment effects in RA. Our approach included estimating cell contributions from gene expression data and comparing it to flow cytometry-derived cell composition, followed by the identification of gene regulatory changes. We hypothesized that unbiased computational approaches^31^ could be used to identify cell contributions and that estimation of cell contributions can be used to identify disease and treatment-related changes.

We identified genes that were correlated with changes in cell compositions and genes that are associated with disease treatment even when cell composition is taken into account. Our analysis suggests that the majority of expression changes are due to changes in cell proportions rather than cell-intrinsic changes in gene expression.

## Patients and methods

### Patient cohort

Our cohort was derived from the COMBINE cohort^32^ which is a comprehensive cohort consisting of multi-omics data of pre- and post-treatment samples from more than 160 RA patients receiving either TNFi or MTX therapy. The cohort included patients with RA, according to the ACR 1987 or the 2010 ACR/EULAR criteria, who were undergoing change or starting a new treatment regimen at the Rheumatology Clinic, Karolinska University Hospital, Stockholm from February 2011 to May 2013.

In our study, we included 53 DMARD treatment naïve early RA patients who started MTX treatment and were able to provide peripheral blood samples before the start of MTX treatment and following a follow-up visit of approximately three months (median 93 days). Patient demographics and baseline disease characteristics are provided in Supplemental Table 1. MTX treatment was given as a monotherapy according to current practice in Sweden and therefore the results are not complicated by effects from other DMARDs. Additionally, our cohort included 37 individuals who previously did not respond to MTX treatment and started TNFi treatment and were able to provide peripheral blood samples before the initiation of treatment and approximately 3 months post-start of treatment.

In addition to RA patients, we collected samples from 30 age-matched healthy controls at two time points separated by approximately 3 months. Only individuals with high-quality RNA samples with both time points were considered for further analysis.

We used the European League Against Rheumatism (EULAR) response criteria to classify patient response to treatment^33^. A qualified rheumatologist at Karolinska University Hospital performed the evaluation of response. In our analysis, we considered Good and Moderate EULAR responders as “responders” and compared these to the EULAR “non-responders”. Our cohort contained 31 MTX responders and 26 TNFi responders.

### Data generation

The RNA was extracted from PBMCs, freshly isolated using CPT tubes (BD Biosciences) using isopropanol extraction and sequenced as previously described^32^. Briefly, RNA was sequenced using an Illumina HiSeq 2000, the TruSeq RNA sample preparation kit with 2 × 100 base pair (bp) paired-end reads to a mean read depth of 15.7 million read pairs per sample. The sequencing reads were trimmed using Trim Galore (http://www.bioinformatics.babraham.ac.uk/projects/trim_galore/) and then mapped to the GRCh38 human reference genome and subsequently gene counts were generated using Star v.2.5.3a^34^.

### Flow cytometry analysis

The Clinical Chemistry laboratory of Karolinska University Hospital measured the absolute numbers of leukocytes, neutrophils, eosinophils, basophils, and monocytes per liter of peripheral blood using XE Sysmex flow cytometry-based analysis. Peripheral blood mononuclear cells (PBMC) were stained freshly using the following antibodies (clones): CD45RA (B56), TcRgd (B1), HLA-DR (L43), CD4 (OKT4), CD138 (ID4 or DL-101), CD19 (HIB19), NKp44 (P44-8), CD16 (3G8), CD69 (FN50), CD28 (CD28.2), CD45 (HI30), IL21R (2G1-K12), TREM-1 (TREM-26) all from Biolegend, CD3 (UCHT1) and NKG2A (Z199.1) from Beckman Coulter, IgD (IA6-2), CD14 (Mphi 9), CD27 (M-T271), CD56 (BI59) from Beckton Dickinson, NKG2D (1D11) from eBioscience. The definition of major cell populations was as described in^35^, and performed at the Division of Rheumatology in the Center for Molecular Medicine, Karolinska Institutet.

### Glucocorticoid signature

We utilized the gene signature by Hu et al.^36^ to estimate the level of exposure to glucocorticoids (GC) in each sample. We used the single-sample gene set enrichment analysis (ssGSEA) algorithm^37^ to calculate the composite score of the enrichment level of GC gene signatures in each individual sample. The Gene Set Variation Analysis package in R was employed which ranks genes in the transcriptome within each sample and scores genes of interest according to the ranks.

### Cell contribution

To extract cell-type specific information from heterogeneous samples, we used the computational deconvolution procedures implemented in CytoPro^38^. To employ this algorithm, cell type markers for blood tissue were previously generated from a collection of sorted cell data from blood, which included 29 cell types from blood or in-vitro assays (e.g. Th1, Th2) from which unique markers were identified which were optimal for cell deconvolution^38^. The cell type contribution score estimates the contribution of a given cell type to the total amount of RNA measured in each sample.

To determine the cell signatures, a manually curated compendium of 9000 samples of bulk gene expression data was used as input to generate and select cell-specific signatures^39,40^. These signatures are then used by a deconvolution method to quantitate per-sample cell content and output cell contribution score, which is a proxy for the cell type proportions in a sample. This score has arbitrary units but is overall correlated to the real cell type proportions. These values are comparable within a given cell type between different samples that were processed and normalized together as part of the same dataset. However, the scores cannot be interpreted as the actual number of cells.

### Effect of conventional treatment on cell contributions in RA patients

To determine the effect of treatment (MTX or MTX + TNFi) on computationally derived cell contributions we used paired samples (pre- and post-treatment) and modeled the association between cells and time (within-subject) in a linear model using the *lm* function in R, as follows:

$$cells \, \sim \, subject \, + \, GC\_signature \, + \, treatment$$

where cells represent the cell contribution score for the 29 cell types evaluated, subject is patient id, GC_signature is the enrichment level of GC gene signatures and treatment is either MTX or MTX + TNFi. To model the difference in treatment effect with respect to response, the binary response was added as an interaction term:

$$cells \, \sim \, subject \, + \, GC\_signature \, + \, treatment \, + \, treatment:response$$

where response corresponds to the EULAR response criteria.

### Effect of conventional treatment on gene expression in RA patients

The effects of treatment (MTX or MTX + TNFi) on gene expression profiles in PBMCs were tested in paired samples (pre- and post-treatment) using the R package limma voom^41^, as follows:

$$gene\sim \, subject \, + \, GC\_signature \, + \, treatment$$

where gene represent the expression value for a specific gene. To model the difference in treatment effect with respect to response, the binary response was added as an interaction term to the model:

$$gene\sim \, subject \, + \, GC\_signature \, + \, treatment \, + \, treatment:response$$

Models were run both with and without adjustment for computationally derived cell contributions (CD4+ alpha-beta T cell, monocyte, plasma cell) as well as adjusted for technical covariate of percent duplicates.

### Prediction of treatment outcome in naïve and established RA patients

We built binary classifiers on the baseline cell contributions data using Elastic Net (as implemented in the R package caret^42^ for predicting non-responders of MTX treatment (47 responders, 13 non-responders, prevalence 21.7%) or TNFi treatment (30 responders, 12 non-responders, prevalence 28.6%). Prior to prediction models, cell contributions were inverse rank normalized (as rank-based features). A nested cross-validation procedure was used to avoid overfitting when optimizing the model. In each repeat, a random set of four-fifths of the samples (“training set”) were used in optimization of the models, and one-fifth of the samples (“test set”) were kept out for testing of prediction performance, making sure to preserve group proportions. The models were optimized in tenfold cross-validation on the training set, keeping the model with the best overall performance in assigning patients to non-responder/responder (based on AUC). The prediction performance of the model was assessed as the ability of the model to predict samples in the test set to non-responder (as assessed by AUC and Precision recall (PR) better suited in cases with a low prevalence of non-responder group). This procedure was repeated 100 times to get a proper assessment of prediction performance.

### Meta-analysis

Data from three public RA studies were downloaded from the Gene Expression Omnibus database^43^ as GEO accession numbers [GEO accession: GSE45291 (n = 493), GSE93272 (n = 232) and GSE90081 (n = 12)]. Statistical integration of cell differences (Meta-analysis) from the different datasets was performed with a random effects model to account for study heterogeneity^44^.

### Statistical analysis

The cell type differences were calculated using the Wilcoxon rank sum test as implemented within R (wilcox.test). The test was employed in paired or unpaired mode depending on the comparison. The P-values were corrected for multiple hypothesis testing using the Benjamini–Hochberg false discovery rate^45^. Gene expression differences were evaluated using limma R package^46^. All analyses were implemented in R version 3.6.2 and all visualizations were created using the ggplot2 R package version 3.3.6^47^.

### Ethics approval and consent to participate

The COMBINE biobank was generated after written informed consent from all participants had been obtained according to the Declaration of Helsinki and with approval by the Stockholm (number 2010-351-31-2) and Uppsala (2009-013) Regional Ethics Committees.

## Results

### Generation of a comprehensive cell contribution dataset for RA

**Figure 1:**
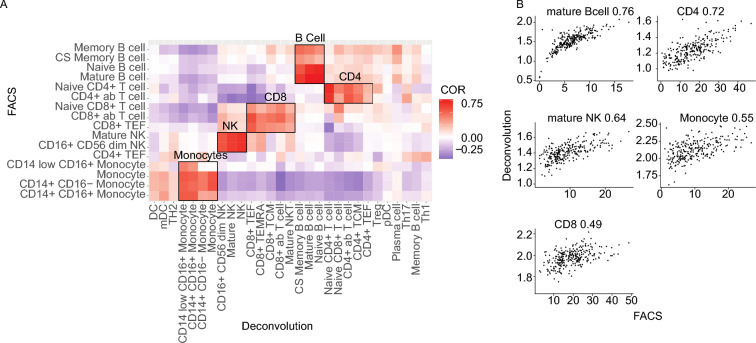
Correlation of flow cytometry to predicted cell compositions from gene expression. (**A**) Correlation heatmap comparing cell proportions derived from flow cytometry and gene expression derived cell compositions in blood of RA patients at baseline. (**B**) Direct comparison between flow cytometry and predicted cell compositions from gene expression for 5 selected cell populations: Mature B cells, CD4+ T cells, Mature NK cells, Monocytes, and CD8+ T cells.

We used a computational deconvolution procedure (CytoPro) to determine the proportions of 29 cell types. To verify the accuracy of CytoPro we compared the predicted cell type contributions per sample to the measured cell proportion determined by flow cytometry (Fig. 1A). We observed a positive correlation between the two methods for 5 major high-level ontology definitions with statistically significant correlation coefficients ranging from 0.49 to 0.76 (Fig. 1B). It is important to note that the flow cytometry data was not used to train or test the deconvolution algorithm. Since immune cell subpopulations were assigned based either on protein markers (flow cytometry) or RNA gene expression (computational method) our methods are not redundant and equally informative for our further analyses.

### Cell changes associated with disease

**Figure 2:**
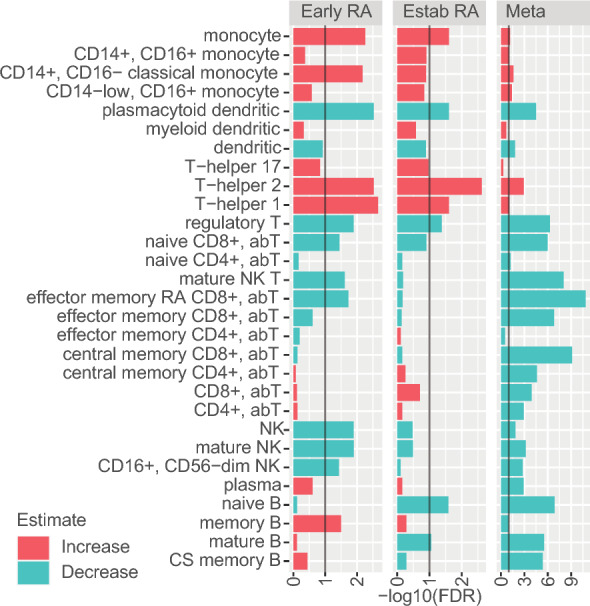
Changes in cell proportions associated with RA disease. Bar plots showing the estimates of the contribution of a given cell type to the total amount of RNA measured in each sample. This score has arbitrary units but is overall correlated with the actual cell type proportions. The values are comparable within a given cell type between different samples that were processed and normalized together as part of the same dataset. The directionality of change (up/down) for each cell type is represented by a color code (green = down, red = up).

We investigated changes in peripheral blood cell types in two cohorts of RA patients, those with early disease who were recently diagnosed with RA and were DMARD treatment naïve and those with established disease whose disease was not adequately controlled by MTX therapy. Using healthy controls as a reference, we found that several cell types in PBMCs of RA patients were significantly altered (Fig. 2). Both classical and non-classical Monocytes and CD4^+^ cells (T_h_1 and T_h_2) were significantly increased (FDR < 0.05) in both early and established RA patients, while NK cells and B cells were significantly downregulated in early and established RA, respectively. Furthermore, B cells showed an increase in early RA with significant changes observed for memory B cells. To confirm our findings, we performed a meta-analysis of cell deconvolved data from three publicly available gene expression datasets^20,48,49^. We found directionally consistent significant cell changes for 12 out of 13 cell types in the meta-analyses (Fig. 2). The meta-analysis showed a decrease in B cells, mirroring the changes in established RA in the COMBINE cohort, likely owing to the fact that most of the publicly available studies focus on established RA subjects. Additionally, the larger sample sizes in the meta-analysis (n = 747), revealed significant changes associated with RA for many cell types that did not show significant changes in our initial analysis.

**Figure 3:**
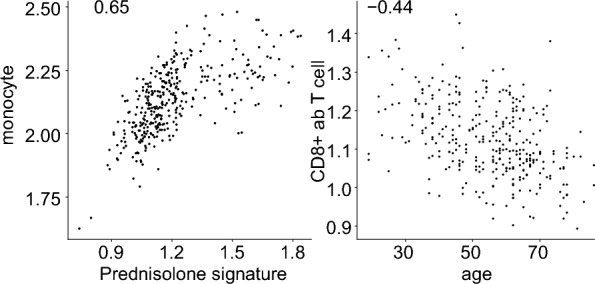
Correlation of baseline clinical variables with cell compositions. (**A**) The calculated monocyte proportions were positively correlated with a gene expression module representing GC treatment. (**B**) The age of RA patients was negatively correlated with the proportion of naïve CD8^+^ ﻿αβT cells.

Given the extensive clinical data available for this cohort, we investigated whether there were any associations between differences in cell contributions at baseline and clinical variables at the time of sampling. Since information on the amount and timing of Glucocorticoid (GC) treatment, which has a dramatic effect on gene expression, was not consistently collected in our study, we used a GC signature as a proxy to GC exposure^36^. Our analysis revealed a strong correlation between GC exposure and monocyte cell proportions (as shown in Fig. 3). Additionally, we observed a negative correlation between CD8^+^ αβT cells and age, but we did not find any significant correlations between cell contributions and disease-related variables such as DAS28 components, pain, or smoking status (data not shown).

### Cell changes correlated with treatment effect

**Figure 4:**
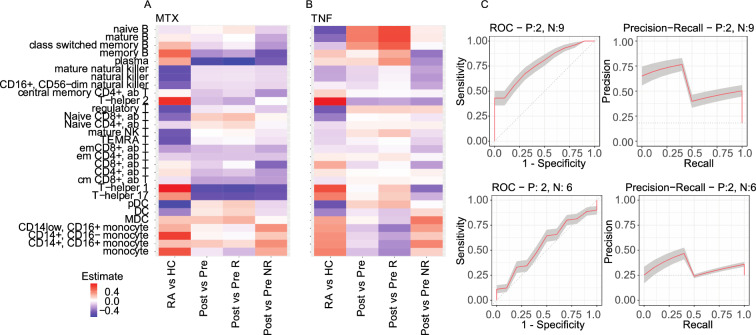
Changes in cell proportions associated with treatment. The heatmap shows the proportion of cell types that change in RA compared to healthy controls (HC). (**A**,**B**) Additionally, the proportions were calculated before and after treatment with MTX and TNFi and for the responder (R) and non-responder populations (NR). The directionality of change (up/down) is represented by a color code (blue = down, red = up, white = no change). (**C**) Receiver operator curves and precision recall curves from predictions algorithms that use cell proportion to predict MTX responders (upper panels) and TNFi responders (lower panel).

In general, treatment with MTX or TNFi led to a reversal of the changes in cell contributions observed when comparing healthy controls and patients with disease (Fig. 4). In the early RA group, treatment with MTX resulted in a decrease in B cells (Memory and Plasma cells) and CD4^+^ T_h_ cells (T_h_1 and T_h_17) but had a limited effect on the monocyte population.

In contrast, treatment with TNFi in established RA patients significantly increased the population of B cells while generally not affecting the population of many other cell types. It is interesting to note that there was a dichotomous change in B cell populations where the proportions of B cells increase in early RA but decrease in established RA compared to healthy individuals without treatment. Both MTX and TNFi tended to reverse changes in B cells back to near healthy-levels. Adjustment for GC treatment only had a marginal effect on changes in cell proportions (data not shown).

### Cell changes correlated with response

We investigated whether changes in cell compositions that occurred after MTX treatment or TNF blockade differed among responders and non-responders.

We found that there were no changes in cell composition in subjects that responded to MTX and a significant decrease in plasma cells in those subjects that responded to MTX (Fig. 4A). PBMCs from subjects that responded to TNFi treatment were significantly associated with an increase in mature B cells and a decrease in monocytes, whereas in non-responding subjects, no statistically significant changes were detected (FDR < 0.1). (Fig. 4B). We noticed that a decrease in mature B cells and an increase in monocytes were significantly associated with RA compared to healthy controls, suggesting that changes in these populations in patients during treatment may be associated with the treatment outcome.

Given the differences in cell changes between responders and non-responders to treatments, we developed prediction algorithms to determine if these differences could be used to predict non-responders to current therapies. We built a classifier to predict treatment non-response from cell contributions at baseline using a logistic regression model and detected response as a binary factor (0 = No response, 1 = Moderate/Good response). We observed predictive performance with a mean AUC = 0.79 [0.44–1.0], and mean PR AUC = 0.59 [0.11–1.00] (Fig. 4C, Table 1) for predicting non-responders to MTX. In an analogous analysis of response to TNFi treatment, we observed lower prediction performance than for MTX in the classification of non-responders (mean AUC = 0.63 [0.33–1.0], mean PR AUC = 0.35 [0.0.13–0.80], Fig. 4C, Table 1). This may be due to the lower number of patients in this sub-stratum.

**Table 1:** Prediction of non-responders.

Treatment	AUC-ROC			Se	Sp	AUC-Precision Recall				Pr	Re
	Mean	Min	Max			Mean	Min	Max	F		
MTX	0.79	0.44	1.00	0.56	0.83	0.59	0.11	1.00	0.48	0.42	0.56
TNFi	0.63	0.33	1.00	0.48	0.52	0.34	0.13	0.80	0.33	0.25	0.48

*Se* sensitivity, *Sp* specificity, *F* F measure, *Pr* precision, *Re* recall.

### Regulated genes after adjustment for cell composition

**Figure 5:**
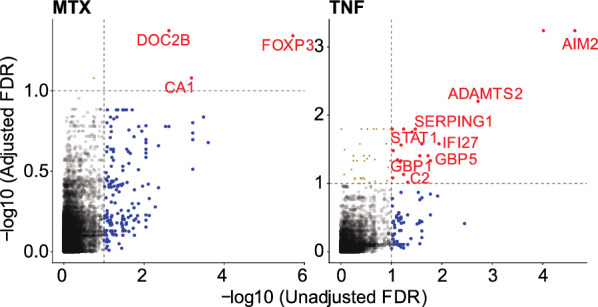
Gene expression changes associated with cell proportions. Scatterplots show the change in -log10 FDR values before and after adjustment for cell type proportions for MTX treatment over baseline (**A**) and TNFi treatment over baseline (**B**). The x-axis represents the FDR values adjusted for a technical variable (% percent duplicates) while the y-axis is adjusted for cell proportions for three main cell types (Monocytes, CD4^+^ T cells, and Plasma cells) and adjusted for prednisolone signature gene sets. The points are colored red if they are significantly regulated both pre- and post-adjustment, blue if they are regulated before cell proportion adjustment and green if they are regulated only after cell type adjustment.

The variability in the proportion of cell subtypes between samples has been shown to strongly contribute to observed differential expression in bulk tissue data^50^. Given the dramatic changes seen in cell composition, we determined the variation in gene expression level by statistically adjusting for sample cells. To achieve this, we compared gene expression changes between treated and untreated paired samples and modeled them with covariates to adjust for both technical variables (% duplicate reads) and biological variables (GC signature). In secondary models, we also included calculated cell contributions for three variables and non-correlated cell types (monocytes, CD4^+^ T cells, and plasma cells) to identify genes modulated by treatment that were not explained by changes in cell contribution. Adjusting for cell contributions accounted for most of the variation in treatment-associated expression (Fig. 5). In MTX-treated subjects, only three genes (FOXP3, CAI1, DOC2B) out of 157 remained significantly differentially expressed after adjustment for cell contributions. While after TNFi treatment 20 genes out of 76 remained significant including genes involved in innate immunity (AIM2, C2, GBP1, GBP5, SERPING1), IL1b- secretion (AIM2, GBP5), and interferon response (IFI27, STAT1, AIM2). We also identified 2 genes for MTX and 32 for TNFi, respectively, that became significant only after adjustment for cell contributions. These include interferon-inducible genes (OAS1, IRF7, IFI35, IFI44, IFI44L) and antigen presentation pathway genes (CD86, HLA-DMA, HLA-DMB, HLA-DPA1, HLA-DPB1, HLA-DRB3) and potentially represent new regulatory signals that are masked by differences in cell contributions.

## Discussion

Rheumatoid arthritis is characterized as an immune-mediated disease and extensive research has been conducted over the years to characterize the molecular changes that occur in patients with RA. Many studies have investigated changes in cell composition^51^ and others have studied the molecular effects^15–21^. In a previous study, we extensively characterized a cohort of RA patients and collected PBMC samples from pre- and post- MTX and TNFi-treated patients (COMBINE study)^32,35,52^. We compared gene expression differences in PBMCs and investigated the proportion of different cell types and cell phenotypes measured using flow cytometry. Our analysis revealed significant changes in a number of immune cell proportions following MTX treatment including an increase in HLA-DR^+^ T cells. In this study, we aimed to determine the interplay between molecular and cellular changes associated with RA. We used a computational deconvolution approach to predict cell type proportions based on bulk RNA expression data and found a high correlation to traditional flow cytometry-based methods. We then used the predicted cell contributions to expand the identification of cell types that change with disease and treatment. Finally, we show that the majority of changes observed by gene expression analysis can be attributed to changes in cell composition, and we also identify disease-associated genes that are independent of changes in cell proportions.

Cell deconvolution is a powerful approach to estimate the relative proportions of different cell types within a mixed cell population based on gene expression data. In the context of RA, which is characterized by chronic inflammation and autoimmunity, cell deconvolution has important implications for understanding disease pathogenesis, identifying novel therapeutic targets, and developing novel treatment strategies.

Chronic inflammation is associated with infiltration of immune cells into the synovial tissue. Different immune cell subsets, such as T cells, B cells, macrophages, and neutrophils, contribute to the inflammatory process and may have distinct roles in disease development and progression^53^. By using cell deconvolution methods to analyze gene expression data from peripheral blood samples of RA patients we estimated the relative proportions of these cell subsets and identified changes in their abundance during disease course.

By identifying specific cell subsets that are dysregulated in RA, cell deconvolution can also help to identify potential therapeutic targets. For example, therapies that target specific subsets of T cells^54^ or B cells^55^ have shown promise in clinical trials.

Here we applied cell deconvolution which is a powerful tool to understanding the role of different immune cell subsets in RA pathogenesis and to identify potential therapeutic targets. Its use can lead to a better understanding of the underlying mechanisms driving the disease and ultimately lead to more effective treatment strategies for patients with RA.

### Disease associated changes in cell composition

We found that both established and early RA were associated with an increase in monocytes (both classical and non-classical) and CD4^+^ cells (T_h_1, T_h_2, and T_h_17). The role of monocytes in RA disease progression is well documented; they are central to the initiation of inflammation^56^, found in sites with high inflammation^57^, produce high levels of TNF^58^, and can lead to bone erosion^59^. Infiltration of CD4^+^ T cells is another hallmark of inflammatory disease and CD4^+^ T cells have been implicated in RA disease. CD4^+^ T cells can produce IFNγ^60^ which activates macrophages^61^ and are involved in B-cell activation.

The B lymphocytes are also involved in many aspects of RA pathogenesis^62^. They are precursors of plasma cells that produce anti-citrullinated protein antibodies and Rheumatoid factors which are hallmark markers of RA. In addition, B cells contribute to T cell activation and can act as antigen-presenting cells promoting immune infiltration. B-cell depletion has been used as a therapy to treat RA patients. In this study, we show that the circulating B lymphocytes decrease in established RA but increase following treatment with TNFi. This is consistent with other reports showing that there is a decrease in B cell populations that returns to near-normal levels following TNFi therapy^63^.

We also observed an increase in NK cells, particularly in the early RA cohort. NK cells are an important part of the innate immune system, acting as a first-line defense during immune challenges and having the ability to kill a variety of target cells^64^. They process cytolytic activity as well as the ability to produce cytokines. The role of NK cells in RA is unclear, but our data suggest that they may play a more critical role in early RA and have a diminished role once disease has been established.

### Genes associated with MTX treatment

We also analyzed the MTX data to elucidate disease-relevant genes that were not solely related to changes in cell contribution. In models adjusted for cell contributions, we identified three genes (FOXP3, CA1, and DOC2B) in our early RA cohort that change with treatment independently of cell-related changes.

Foxp3+ regulatory T cell (Treg) is a major immune cell suppressor and we and others^65^ have shown suppression of these cells in RA patients. However, we found the expression of FOXP3 was not correlated with the proportion of T-reg cells. In this study, we found an increase in FOXP3 expression in early RA, while the proportion of T-reg cells is decreased, suggesting that FOXP3 may have an independent function in RA pathogenesis.

Carbonic anhydrase I (CA1) is involved in the process of bone formation and is indicated in susceptibility to ankylosing spondylitis and therefore may be important for bone erosion seen in RA. We found that CA1 expression was decreased in early RA patients.

### Genes associated with established RA

Our analysis identified twenty genes associated with treatment in established RA, which were independent of changes in cell composition. Many of these genes have been associated with processes involved in RA progression. This included several genes associated with type 1 interferon response^66^ (GBP1P1, IFI27, IFI44L, UFI44, GBP4, GBP5, LGALS3BP, SMAD4A). Interferon response is mediated by multiple cell types and has been associated with multiple rheumatic diseases^66^. Additionally, several disease-associated genes were implicated in immune-mediated functions such as AIM2 (Absent in melanoma 2), which activates inflammasome formation in macrophages in response to double-stranded DNA and GBP5 (Guanylate Binding Protein 5) which is upregulated in synovial fibroblasts. GBP5 knockout studies in animals exacerbates disease and increases bone destruction^67^. TG2 (Transglutaminase 2) is associated with wound healing and inflammatory diseases^68^ and linked with cartilage degradation^69^.

Recent studies have explored the differences of TNF between responders and non-responders using individual transcripts^70^ or expression modules^71^. However, many of these studies had limited ability to differentiate between responders and non-responders, resulting in a low predictive ability to predict responders. Moreover, there is a relatively low overlap of DEGs identified in previous studies of TNFi^72^. This may be due to patient heterogeneity, which could make it difficult to accurately detect differentially regulated genes. Alternatively, differences in cell abundance could contribute to patient heterogeneity. More work needs to be done to determine if differences in cell abundance can explain the observed patient heterogeneity in most studies.

Despite these findings, there are some limitations to our study. We used cell markers that were partially derived from healthy individuals, which may have missed some changes in cell state due to disease and/or treatment. In addition, our method was limited to a predefined set of cell types and could not identify potential novel cells or cell states. While newer single cell technologies would allow us to capture such novel cell types and cell states, our method has the distinct advantage to be able to utilize historical bulk RNAseq data in particular where methods to determine cell contributions were not employed. Finally, we relied on cell signatures derived from blood to learn about RA, even though the main site of the disease is in the synovium. It is worth noting that changes in cell proportions in the blood may differ from changes in the synovium.

## Conclusions

In summary, our computational approach to identifying cell contributions has allowed us to determine that the majority of genes regulated in rheumatoid arthritis are not directly associated with regulatory changes of genes, but rather associated with changes in cell proportions. We have also identified several disease-associated genes that warrant further exploration as potential targets or biomarkers of disease progression. We applied prediction algorithms to determine if the cell proportion can be used to predict response to the current standard of care therapy and while these results are encouraging, we plan to extend our studies into larger patient cohorts to validate these findings. We believe that the methods we employed can be applied to other diseases to better understand the contributions of cell proportions to disease and to deconvolve the overall gene expression signature.

## Supplementary Information

Supplementary Table 1.

## Data Availability

Processed data have been deposited to NCBI's Gene Expression Omnibus (GEO) under the GEO accession number GSE229449 https://www.ncbi.nlm.nih.gov/geo/query/acc.cgi?acc=GSE229449. The code used to process the RNA sequencing files, binary classification methods, and figure generation is now publicly available at https://github.com/pfizer-opensource/Peripheral-blood-cellular-dynamics-of-Rheumatoid-arthritis. The raw datasets generated and analyzed during the current study are not available in a public repository according to the ethics permissions for COMBINE. Please contact author LP with data requests for applicable studies.

## Abbreviations

MTX: Methotrexate
TNFi: Tumor necrosis factor inhibitor
EULAR: European Alliance of Associations for Rheumatology
RA: Rheumatoid arthritis
PBMCs: Peripheral blood mononuclear cells
NK: Natural killer
DMARDs: Disease modifying drugs
csDMARDs: Conventional synthetic disease modifying drugs
tsDMARDs: Targeted synthetic disease modifying drugs
FLS: Fibroblast-like synoviocytes
GWAS: Genome-wide association study
scRNA-seq: Single cell RNA sequencing
CPT: Cell preparation tube
GC: Glucocorticoids
ssGSEA: Single-sample gene set enrichment analysis
AUC: Area under the curve
PR: Precision recall
GEO: Gene Expression Omnibus
