# Supplementary material for: Peripheral blood cellular dynamics of rheumatoid arthritis treatment informs about efficacy of response to disease modifying drugs
Source: Sci Rep. 2023 Jun 21;13:10058. doi: 10.1038/s41598-023-36999-0 (PMC10284888; doi:10.1038/s41598-023-36999-0)
Supplement: Supplementary file 1 — Supplementary Table 1. [file 41598_2023_36999_MOESM1_ESM.docx]

Supplemental Table 1: Patient demographics and baseline disease characteristics

| Category | Levels | Value |
| --- | --- | --- |
| Age | Mean (SD) | 57.4 (13.9) |
| Sex | Female | 64 (69.6%) |
|  | Male | 28 (30.4%) |
| Ethnicity | Swedish | 74 (80.4%) |
|  | European | 6 (6.5%) |
|  | Non-European | 11 (12.0%) |
|  | (Missing) | 1 (1.1%) |
| DAS28 | Mean (SD) | 4.8 (1.2) |
| CRP | Mean (SD) | 9.7 (12.9) |
| ESR | Mean (SD) | 24.6 (16.7) |
| RF | negative | 42 (45.7%) |
|  | positive | 49 (53.3%) |
|  | (Missing) | 1 (1.1%) |
| Anti_CCP | negative | 30 (32.6%) |
|  | positive | 61 (66.3%) |
|  | (Missing) | 1 (1.1%) |
| Seropositive | Negative | 44 (47.8%) |
|  | Positive | 47 (51.1%) |
|  | (Missing) | 1 (1.1%) |
| Swollen_joints | Mean (SD) | 7.4 (5.4) |
| Tender_joints | Mean (SD) | 7.4 (5.7) |
| DA_Score | Mean (SD) | 41.9 (16.6) |
| Erosion | erosions | 35 (38.0%) |
|  | none | 56 (60.9%) |
|  | (Missing) | 1 (1.1%) |
| MEDs | Cimzia | 2 (2.2%) |
|  | Enbrel | 8 (8.7%) |
|  | Humira | 14 (15.2%) |
|  | None | 54 (58.7%) |
|  | Remicade | 13 (14.1%) |
|  | Simponi | 1 (1.1%) |

DAS28: Disease activity score for 28 joints; CRP: C-reactivive protein; ESR: erythrocyte sedimentation rate; RF: Rhuematoid factor; Anti_CCP: cyclic citrullinated peptide antibodies: DA_Score: Disease activity score
